# Visualization and Control of Quasi‐Monoenergetic Proton Shock Waves in a Single Nanoplasma

**DOI:** 10.1002/nap2.70198

**Published:** 2026-07-05

**Authors:** Zhijie Yang, Fenghao Sun, Guangqi Fan, Jinmei Zheng, Hui Li, M. F. Ciappina, Qingcao Liu

**Affiliations:** ^1^ School of Physics Harbin Institute of Technology Harbin China; ^2^ College of Science Harbin Institute of Technology Weihai China; ^3^ School of Information Science and Engineering Harbin Institute of Technology Weihai China; ^4^ School of Marine Science and Technology Harbin Institute of Technology Weihai China; ^5^ State Key Laboratory of Precision Spectroscopy East China Normal University Shanghai China; ^6^ Department of Physics Guangdong Technion Israel Institute of Technology Shantou Guangdong China; ^7^ Technion—Israel Institute of Technology Haifa Israel; ^8^ Guangdong Provincial Key Laboratory of Materials and Technologies for Energy Shantou China

**Keywords:** femtosecond laser pulse, laser‐induced nanoplasma, nanoplasma shock waves, particle‐in‐cell simulation, proton momentum distribution

## Abstract

This work demonstrates direct visualization and deterministic control of quasi‐monoenergetic proton shock waves from isolated gold nanoparticles under intense femtosecond laser irradiation. Using single‐shot nano‐velocity map imaging, we observe a continuous circular shock wave profile in momentum space, reflecting the formation of a symmetric proton shell in space. Two‐stage simulations, combining a modified particle‐in‐cell method with a dissipative Gross–Pitaevskii equation, reveal their formation and expansion on femtosecond–picosecond timescales. Crucially, the proton shock wave energy can be modulated by tuning the laser intensity. These findings provide fundamental insights into strong‐field nanoplasma dynamics and establish a practical scheme for compact, laser‐driven ion sources with on‐demand energy tunability.

## Graphical abstract

Femtosecond laser pulses irradiate isolated gold nanoparticles to yield tunable quasi‐monoenergetic proton shells. Proton energy correlates linearly with laser intensity, providing a compact scheme for easily modulated laser‐driven ion sources.

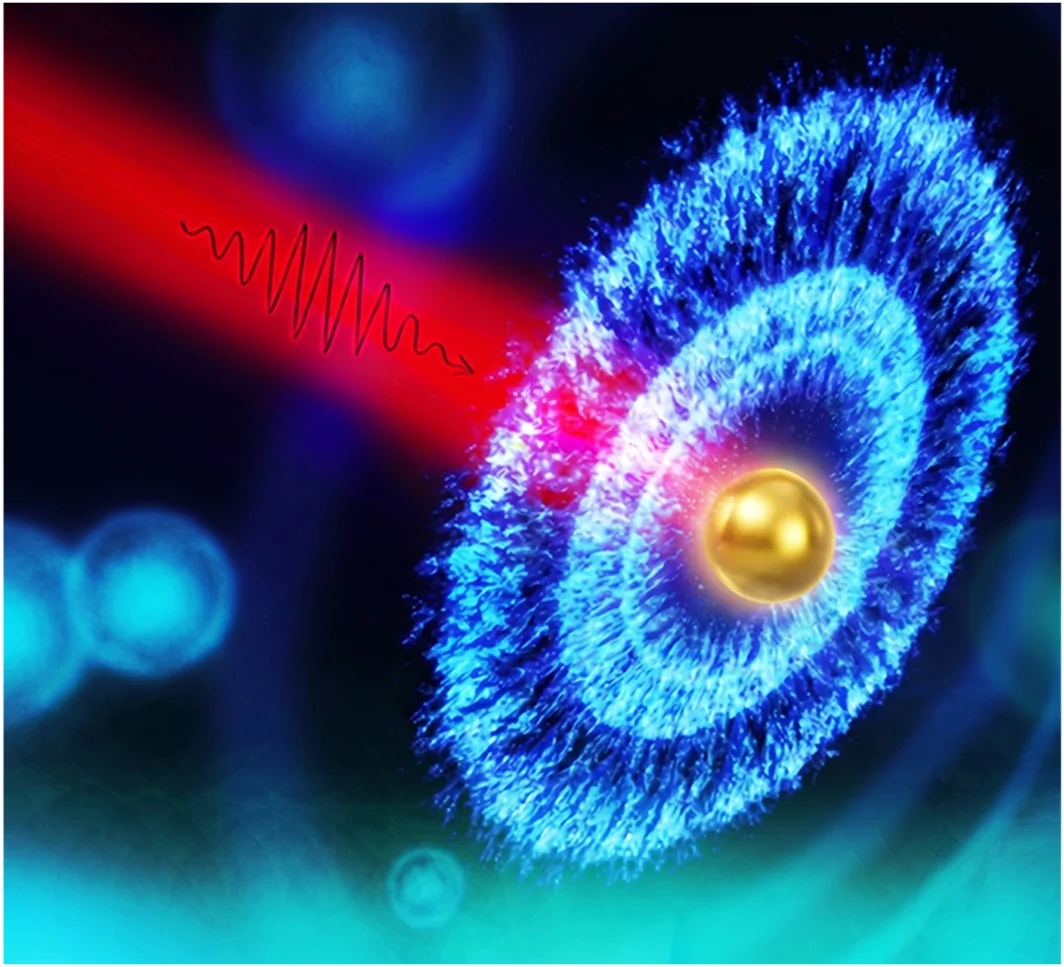

## 1 Introduction

Upon irradiation by intense femtosecond laser pulses, nanoparticles rapidly transition into high‐density nanoplasmas through a sequence of tightly coupled electronic and ionic responses [1, 2, 3, 4]. These laser‐induced nanoplasmas provide a unique laboratory environment for exploring the nonequilibrium and nonlinear processes under extreme multi‐field coupling [5, 6, 7]. Under certain conditions, nanoplasmas can further develop transient shock waves featuring steep density gradients [2, 8, 9, 10]. Here, the term “shock waves” refers to rapidly propagating, sharply localized collective ion/proton fronts generated during nanoplasma expansion in vacuum, rather than the conventional pressure‐driven shock waves in condensed matter physics. The formation and propagation of nanoplasma shock waves occur on transient temporal and nanometric spatial scales, offering a unique window for studying mesoscale physical processes on ultrafast timescales. Moreover, the precise control of nanoplasma shock waves represents an advanced level of manipulation and holds broad application prospects, ranging from precision nanofabrication [11, 12] and compact electron accelerators [13, 14] to targeted oncology treatments [15, 16]. However, current methodologies for directly observing nanoplasma shock waves are hindered by laser‐induced damage to solid targets, leaving significant gaps in the understanding of their formation and evolutionary dynamics. In this context, the combination of precision optical field control with reproducible nanoparticles offers a highly feasible pathway toward advancing the control of nanoplasma shock waves [2, 3, 10, 17, 18, 19].

In this work, we employ single‐shot nano‐velocity map imaging to achieve direct visualization and control of quasi‐monoenergetic proton shock waves induced by femtosecond laser irradiation of gold nanoparticles. By systematically tuning the light‐nanoparticle coupling conditions, the energy of the proton shock waves can be modulated within the investigated intensity range. The underlying dynamics are well reproduced by a modified particle‐in‐cell (PIC) method [20] combined with a dissipative Gross–Pitaevskii equation (GPE) simulation, which can model the nanosystem ionization and the postirradiation nanoplasma evolution [21]. Our simulations predict a linear growth of the final momentum of shock waves with laser intensity in the tested range, consistent with the experimentally observed proportional increase in the radius of the final momentum ring. These findings provide valuable insights into the ionization mechanisms of nanoplasmas under strong‐field excitation and pave the way for further investigations into laser‐induced isolated nanoplasmas.

## 2 Results

Single‐shot momentum imaging of gold nanoparticles was performed using a vacuum experimental apparatus (Figure 1), enabling the selective detection of specific ions ionized from isolated nanoparticles. Aerosolized nanoparticles with diameters of approximately 100 nm were delivered to the high‐vacuum interaction region via an aerodynamic lens, ensuring that each laser pulse interacted with a continuously refreshed nanoparticle. Excitation was driven by linearly polarized laser pulses (50 fs, 1 kHz) with a central wavelength of 800 nm. The laser‐induced ionization of surface molecules triggers the emission of diverse ion species. Owing to their low mass, protons (H^+^) are promptly ejected, preceding heavier ions and thereby serving as ideal diagnostic probes for investigating early‐time plasma dynamics. It should be noted that the detected protons do not originate from the gold nanoparticle itself, but rather from adsorbed surface contaminants (e.g., residual water molecules or sodium citrate molecules) on the gold nanospheres. Through time‐of‐flight analysis combined with high‐voltage gating, protons originating from the tightly bound molecular layer were individually distinguished. Furthermore, two‐dimensional projections of proton momentum distributions (PMDs) were collected using a velocity map imaging (VMI) spectrometer. Detailed descriptions of the experimental setup are provided in the experimental section. Unless otherwise stated, atomic units (a.u.) are used throughout this article.

**FIGURE 1:**
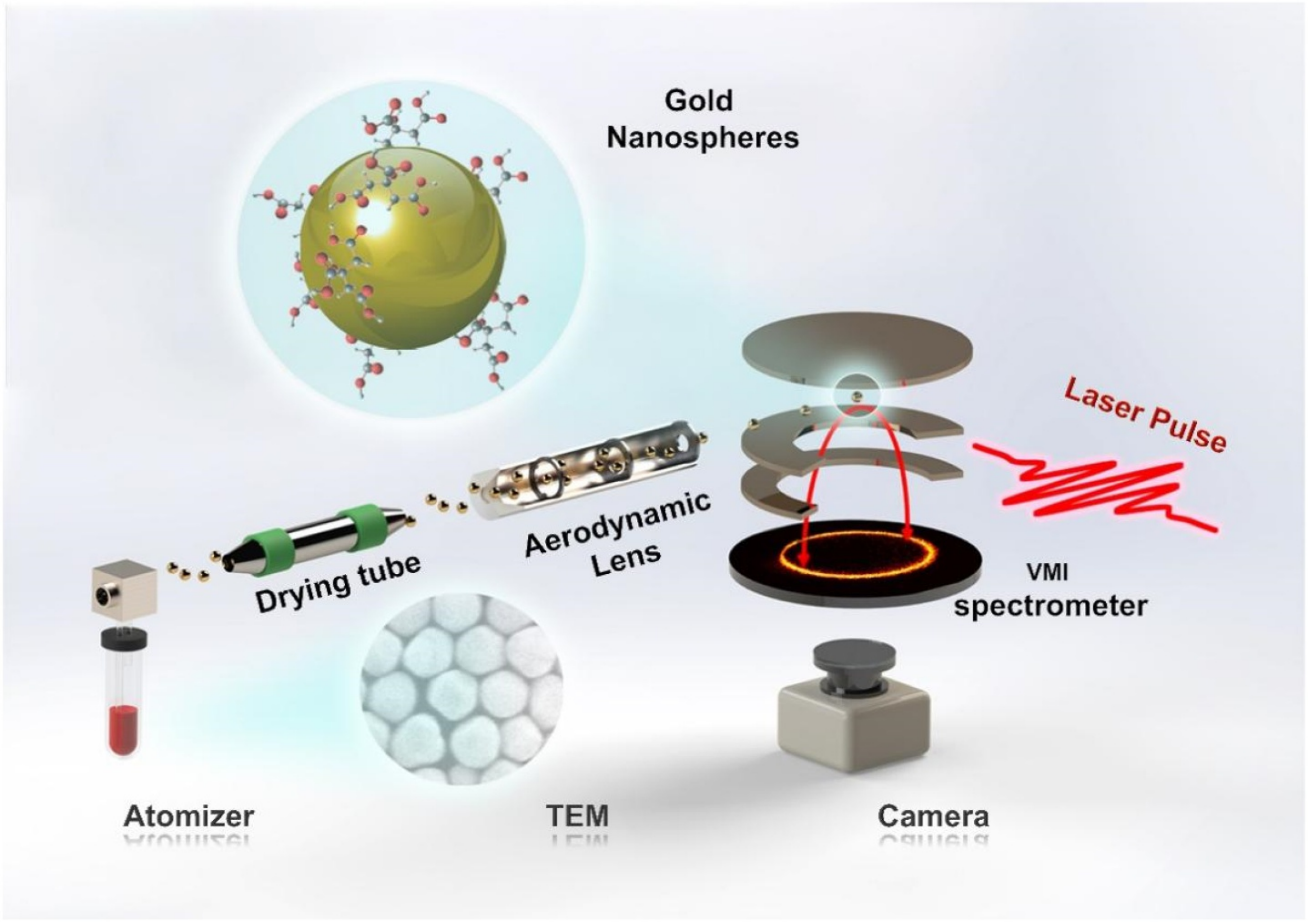
Schematic of a VMI spectrometer apparatus for momentum measurement of nanoparticle ions in a strong field. After atomization and drying, the nanoparticles are focused by an aerodynamic lens into a high‐vacuum chamber, where they are excited by a linearly polarized femtosecond laser pulse. The resulting ion momentum distributions are projected onto two‐dimensional planes using three electrodes inside the VMI chamber and recorded by a high‐speed camera. The inset at the bottom shows a transmission electron microscope (TEM) image of the 100‐nm gold nanoparticles.

Here, single‐shot images are presented to highlight the random asymmetric momentum distribution generated by the interaction between nanoparticles and the laser pulse, with the data analysis detailed in Supporting Information S1 [10, 22]. As shown in Figure 2a, the PMD at a laser intensity of 4.0 × 10^14^ W/cm^2^ manifests as a typical shock wave profile with a sharp density gradient [2, 10]. Notably, the ridge of the shock wave forms a continuous, unbroken circular ring, a feature that contrasts with observations in previous reports [2, 10]. The defect in the top side of the momentum image stems from spatial nonuniformity in the detection efficiency. Statistical analysis of peak radial momenta (Figure 2c), extracted via polar transformation of single‐shot images (Figure 2b), shows that most shock waves manifest as quasi‐monoenergetic features with significant energy localization. Such features stem from the sensitive dependence of the shock wave structure and energy on laser‐induced ionization conditions, where the high‐intensity laser focal region ensures a highly uniform excitation of the gold nanoparticles. Furthermore, we did not detect gold ion signals in our TOF measurements (Figure 2d). This is attributed to the temporal dispersion resulting from the large mass of gold ions (m/q = 197). As indicated by our TOF data, flight‐time broadening is already significant even for sodium ions (m/q = 23), reaching approximately 301 ns, which is much larger than the 78 ns time broadening of protons. For substantially heavier gold ions, the arrival times broaden so extensively that the signal intensity fails to concentrate within a narrow time window, making it impossible to distinguish a clear peak from the background signal.

**FIGURE 2:**
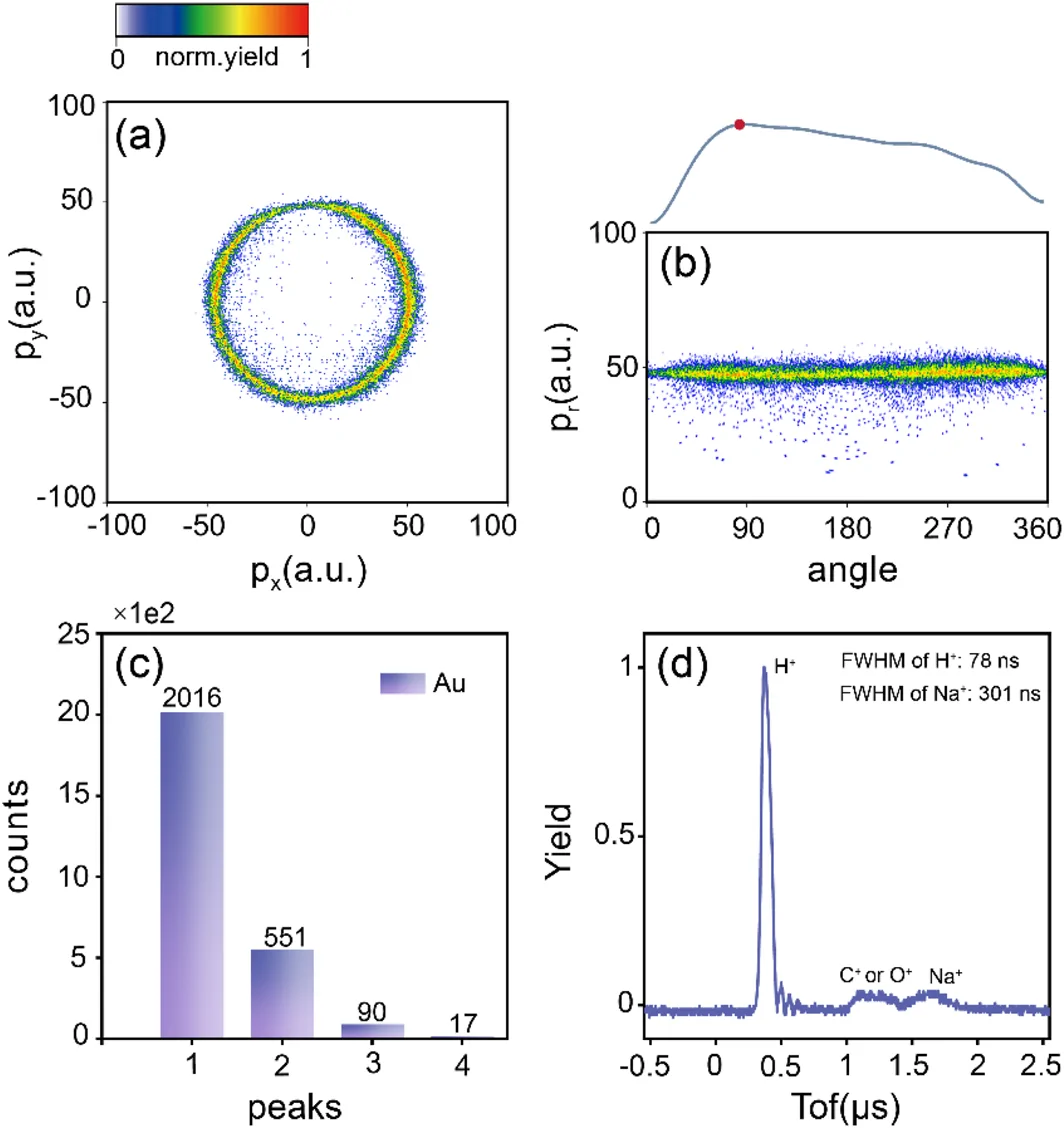
(a) Original single‐shot images (i.e., without any post‐processing such as masking, smoothing, or filtering) of gold nanoparticles under a particular set of conditions (fixed peak laser intensity and nanoparticle size). (b) Momentum distribution in polar coordinates, with the upper inset illustrating the curve of the radial momentum integration. (c) Histogram of the peak number for all single‐shot images. (d) Time‐of‐flight spectra of diverse ion species originating from surface molecular ionization.

## 3 Discussions

Intense laser irradiation triggers the rapid ionization of surface‐adsorbed molecules, creating a hot, dense nanoplasma shell. To further elucidate the formation and subsequent expansion of this nanoplasma, we systematically investigated its ultrafast dynamics over a 100‐ps time window, as illustrated in Figure 3. The calculated spatial distributions of protons at 23.32, 24.22, 38.12, and 50.22 fs are presented in Figure 3a2–d2. Four distinct ionization stages are identified, namely (1) at 23.32 fs (Figure 3a2), protons primarily localize on the upper and lower regions of the nanoparticle's backside. This pronounced back‐ionization, directed along the laser propagation axis, is consistent with the near‐field focusing on the backward side during this phase of the optical cycle (Figure 3a1). (2) After approximately 0.9 fs, both the near‐field (Figure 3b1) and the resulting protons distribution shift from the backward (−*x*) to the forward (+x direction), with the exit angles of the protons gradually converging toward the forward direction (Figure 3b2). (3) The near‐field hot spot (Figure 3c1) and proton emission revert to the backward direction, with the exit angles reconverging on the backside (Figure 3c2). (4) Under a sufficiently high laser field, the near‐field enhancement becomes dominated by the external laser field, localizing along the laser's polarization axis (Figure 3d1). As a result, a dense proton shell forms on the particle surface, exhibiting a smoothly decaying density profile from the core to the vacuum (Figure 3d2). As the driving laser is strong enough to trigger extensive surface ionizations, the near‐field enhancement is dramatically altered on nanometer and femtosecond spatiotemporal scales (Figure 3a1–d1), thereby governing the emission pattern of ionized protons (Figure 3a2–d2).

**FIGURE 3:**
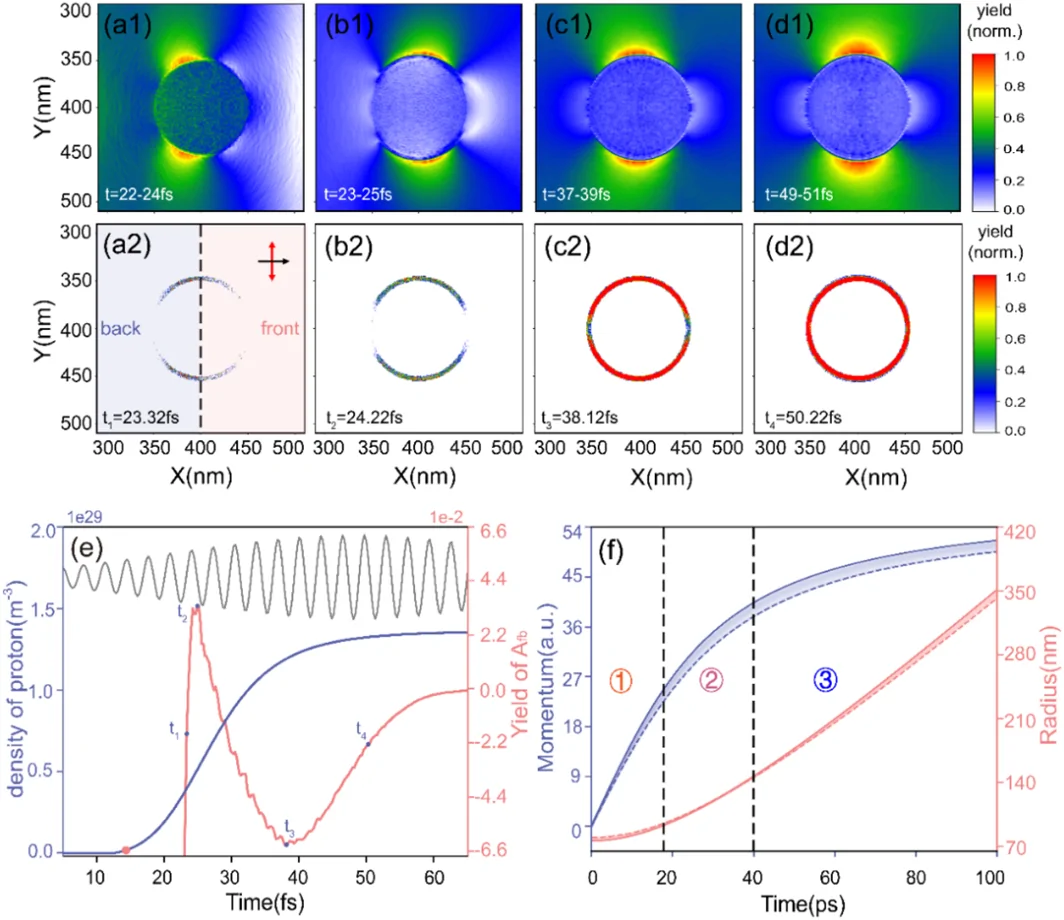
(a1–d1) Calculated averaged distributions of the enhanced electric field around the nanoparticle for the period of 22–24 fs, 23–25 fs, 37–39 fs, and 49–51 fs. (a2–d2) Instantaneous near‐field spatial distributions of protons calculated using PIC algorithm for gold nanospheres at 23.32, 24.22, 38.12, and 50.22 fs. In (a2), the blue and red regions evenly dividing the space indicate areas of backward‐ and forward‐emitted protons, respectively. (e) The density of protons (blue line) and the asymmetry parameter of backward and forward yields (red line) are plotted as functions of time. The time points t_1_, t_2_, t_3_, and t_4_, marked on the red line, correspond to the moments shown in (a2–d2). The gray curve at the top represents the external laser pulse, which oscillates periodically over time. (f) The momentum (blue line) and radius (red line, representing the distance to the nanosphere center) of random protons during 100 ps are plotted. Solid and dashed lines illustrate the flight trajectories of protons from the innermost and outermost parts of the dense proton shell, respectively.

To quantify the emission anisotropy, we integrated the proton yields in the backward (*Y*_b_) and frontward (*Y*_f_) regions. The asymmetry parameter, defined as *A*_fb_ = (*Y*_f_ − *Y*_b_)/(*Y*_f_ + *Y*_b_), is plotted as a function of time (red line, Figure 3e). Due to laser propagation along the +*x* direction, molecules on the nanoparticle's backside are irradiated first, leading to dominant backward ionization in the initial stage. As the laser pulse traverses the gold nanosphere, proton emission transitions rapidly from backward to forward, driven by near‐field enhancement under lower intensity excitation. This optical response of the near‐field region can be classically described by Mie theory [23, 24]. At higher intensities, backward emission again surpasses forward emission due to plasma shielding and localized resonance effects [25]. To provide a detailed analysis of the evolution of plasma, we inspect the number density of generated protons $n_{p}$, which is plotted in Figure 3e as a blue curve. At *t* = 14.35 fs, the number density reaches $n_{p}=$ 1.81 × 10^27^ m^−3^, indicating that the system has entered an overdense plasma state ($n_{p}\;>\;n_{c},n_{c}\approx 1.7\times 10^{27}\;m^{-3}\;\text{at}\;800\;\text{nm})$. This results in significant proton accumulates on the backward side, which blocks the laser propagation and triggers strong localized resonance absorption. Finally, as the laser reaches its peak intensity, the forward and backward yields equalize and $n_{p}$ plateaus, signifying the completion of the excitation and a stable saturation state.

To investigate the long‐term expansion dynamics, representative proton trajectories within the dense shell were tracked, with their radial positions and momenta over a 100‐ps time window plotted in Figure 3f. The evolution exhibits three distinct regimes: Region I: Protons initially closer to the core (solid lines) experience stronger coulomb acceleration from the spatial electrostatic field than those in the outer layer (dashed lines). This gradient in the force field causes the inner protons to accelerate more rapidly, effectively chasing the outermost protons; Region II: The inner protons eventually overtake the outer ones in coordinate space. This trajectory crossing leads to the formation of a critical surface, characterized by a high local charge density, which serves as the precursor to a monoenergetic proton shell. Region III: The accelerated dense shell eventually stabilizes into a monoenergetic ion layer. In this stage, the proton momenta converge toward a specific value (*P* ≈ 50 a.u.), representing the final state of the ion expansion in the far field.

Finally, to investigate the dependence of the proton shock wave on laser intensity, additional experiments were conducted across varying intensities. Figure 4a presents the integrated proton energy spectra for three excitation levels (*I*_0_ = 1.0 × 10^14^ W/cm^2^). The spectra exhibit a narrow profile, with peak energies of 11.79, 16.81, and 21.16 eV, corresponding to a peak momentum of approximately 39.93, 47.70, and 53.45 a.u., respectively. The corresponding proton velocities are estimated to be about $4.77\times 10^{4}$, $5.72\times 10^{4}$, and $6.31\times 10^{4}$ m/s. The associated cutoff energies are 14.75, 20.90, and 25.49 eV. Here, the cutoff energy is defined as the energy on the high‐energy side at which the normalized yield drops to 1% of its peak value. As the laser intensity increases, both the peak energy and the cutoff energy shift toward higher values, and the corresponding peak momentum also increases, indicating that the proton shock wave is highly sensitive to the driving laser intensity. The theoretically calculated peak momenta of dense proton shells, plotted as blue triangles in Figure 4b, reveal a discernible linear dependence on driving intensity. This scaling was rigorously validated by experimental measurements (purple dots in Figure 4b), which showed excellent agreement with the theoretical predictions. Within the investigated intensity range, this behavior can be well described by the empirical linear relation P=k×I+C, where P is the peak momentum, and I is the laser intensity, with fitting parameters *k* = 4.83*I*_0_, and C = 32.87.

**FIGURE 4:**
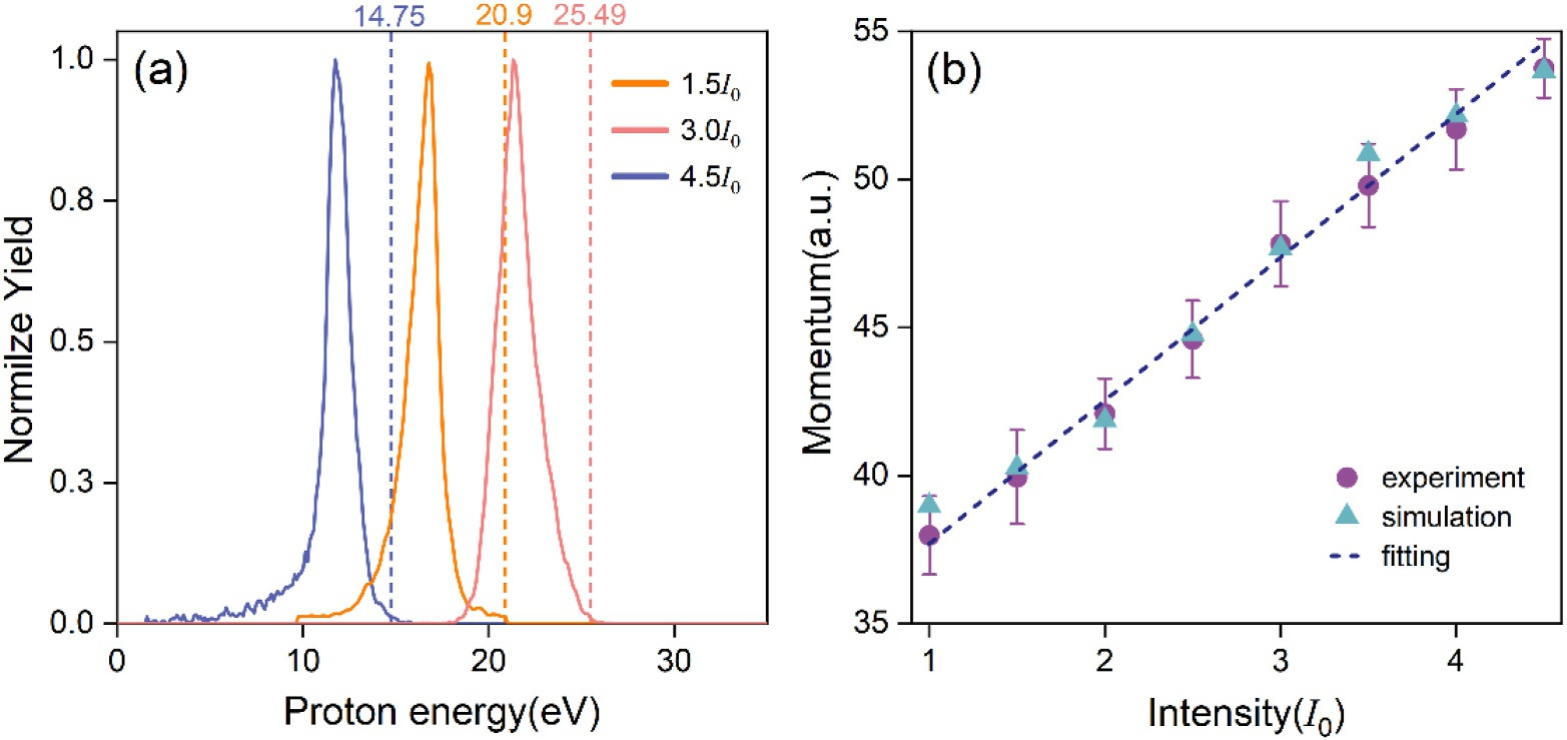
Experimental and theoretical results of proton energy spectra and peak momentum for various excitation laser intensities. (a) Single‐shot integrated proton energy spectra for gold nanospheres at intensities of 1.5*I*_0_, 3.0*I*_0_, and 4.5*I*_0_ (*I*_0_ = 1.0 × 10^14^ W/cm^2^) are shown as blue, orange, and red curves, respectively. (b) Peak proton momentum corresponding to the spectral peak energies are shown as purple dots, with error bars representing the calculated standard deviation. The theoretical values are indicated by blue triangles, and the dashed line represents a linear fit to both experimental and theoretical results.

## 4 Conclusions

We have demonstrated the generation of a highly isotropic, quasi‐monoenergetic proton shock wave from intense laser irradiation of isolated gold nanoparticles, observed directly via single‐shot momentum imaging as a continuous circular shock‐wave profile. By combining experiment with dynamics simulations, a multistage formation mechanism governed by a complex interplay among Mie‐like near‐field enhancement, dynamic plasma shielding, and localized resonance absorption was revealed. This interplay gives rise to asymmetric proton emission in the early stages, which ultimately converges into a dense, symmetric shell in space. Long‐term simulations show that the shell subsequently expands via coulomb explosion, with an ion‐overtaking process generating a stable, monoenergetic layer in the far field. Crucially, the final proton energy exhibits a robust, continuously tunable linear dependence on laser intensity, validated by both theory and experiment. Our findings not only provide fundamental insights into ultrafast nanoplasma dynamics and strong‐field light–matter interactions, but also establish a practical scheme for compact, laser‐driven ion sources with on‐demand energy tunability. Furthermore, appropriate surface modification holds significant potential for increasing the density of launchable molecules while preserving efficient proton shock coupling, thereby providing a new scheme for regulating the proton shock flux.

## 5 Methods

### 5.1 Experimental Setup

The experiment was conducted using a velocity map imaging (VMI) apparatus within an ultrahigh‐vacuum chamber (pressure better than 10^−8^ mbar) [26], where the interaction of a femtosecond laser pulse with individual nanospheres was studied, as shown schematically in Figure 1. These gold nanoparticles exhibited excellent size uniformity, with average diameters of approximately 100 nm, were synthesized using a seed‐mediated growth technique and diluted to 0.01 g/L in pure water.

The nanoparticle colloidal suspensions were aerosolized using a commercial collision‐type atomizer (TSI, Model 3076) with N_2_ gas at 1.5 bar [27]. The aerosolized particles were subsequently dried in a dryer tube (MD‐700 Nafion Dryer, Perma Pure LLC) to remove residual water [28]. A collimated beam of individual nanoparticles was focused into the interaction region using an aerodynamic lens system, while larger aggregates were removed by an impactor unit.

As illustrated in Figure 1, a femtosecond laser pulse generated by a Ti:sapphire amplification system (50 fs, 800 nm, 1 kHz) was directed onto the nanoparticle beam using a concave mirror (*f* = 500 mm). The maximum pulse energy used in the experiment was 0.2 mJ. The focal spot size at the interaction region was estimated to be approximately 40 μm in diameter, resulting in a peak on‐target intensity of up to ∼5.0 × 10^14^ W/cm^2^. The peak intensity of the linearly polarized laser field in the interaction region was estimated by calculating the momentum shifts of above‐threshold ionization (ATI) photoelectrons from Xe gas along the polarization direction [29, 30].

Protons generated from the laser–nanoparticle interactions were accelerated by an electrostatic field onto a microchannel plate (MCP) detector coupled with a phosphor screen. The resulting fluorescence signals were recorded by a high‐speed complementary metal–oxide–semiconductor (CMOS) camera with 10 μs exposure time at a 1 kHz repetition rate. The short exposure was essential to suppress background noise and capture transient ion signals, given the tens‐of‐nanosecond fluorescence lifetimes of both ions and phosphor. A fast high‐voltage gating system, synchronized with the laser pulse, enabled time‐of‐flight discrimination of ion species, providing mass‐to‐charge resolved detection.

### 5.2 Numerical Simulations

Tracking the expansion dynamics of nanoplasmas across the femtosecond‐to‐picosecond regime presents a formidable challenge, as it requires capturing complex nonlinear interactions among numerous charged particles while maintaining high spatial and temporal resolution. To address this, we implement a dual‐stage computational strategy: First, the strongly coupled laser–nanoparticle interaction is modeled using the PIC approach to extract the spatiotemporal distributions of charged particles and self‐consistent fields; this is followed by a dissipative GPE framework to model the postirradiation relaxation of the nanoplasma.

The PIC simulations are conducted using the open‐source two‐dimensional PIC code Smilei v5.0, which incorporates both field ionization (tunneling ionization) and collisional ionization processes for atoms [31]. The simulation box measures 1.024 μm × 1.024 μm with a grid resolution of 1 nm. A 100 nm‐diameter nanosphere is positioned at the center of the simulation box, with the gold nanoparticle core initialized to electron and Au^+^ ion densities of $6\times 10^{28}\;m^{-3}$, corresponding to the bulk density of gold at room temperature [17]. To mimic realistic environments in which nanoparticles are surrounded by solvents or molecular ligands, a 5 nm‐thick neutral atomic shell composed of H and O atoms (2:1 ratio) is introduced, with its ionization energies set to match those of water molecules [17, 19].

In GPE approach, the plasma is treated as a quantum fluid described by a complex‐valued wave function. The governing equation is expressed as

(1) $$i\frac{\partial \psi \left(r,t\right)}{\partial t}=\left(1+i\lambda \right)\left(\frac{p^{2}}{2m}+g{\left|\psi \left(r,t\right)\right|}^{2}+V\left(r\right)\right)\psi \left(r,t\right),$$

where the nonlinear term *g*|*ψ*|^2^ accounts for plasma expansion, the coulomb potential *V*(*r*) represents electrostatic effects, and the dissipative term *iλ* represents environmental energy loss. This equation is solved numerically using a split‐step Fourier method for wave function evolution. Local momentum densities were derived from the spatial gradient of the wave function's phase, and momentum space distributions are constructed via statistical analysis. This method enables efficient and stable simulation of coherent plasma dynamics over femtosecond‐to‐picosecond timescales, capturing expansion behavior beyond the reach of PIC simulations.

## Author Contributions

**Zhijie Yang:** conceptualization, supervision, validation, writing – review and editing, funding acquisition. **Fenghao Sun:** conceptualization, supervision, validation, writing – review and editing, funding acquisition. **Guangqi Fan:** formal analysis. **Jinmei Zheng:** formal analysis, validation. **Hui Li:** writing – review and editing. **M. F. Ciappina:** methodology, writing – review and editing. **Qingcao Liu:** resources, project administration, funding acquisition.

## Funding

This work was supported by the Innovation Program for Quantum Science and Technology (Grant 2024ZD0300700); the National Natural Science Foundation of China (Grants 12204132, 12304376, 12422412, 12227807, 12574092); the Shandong Provincial Natural Science Foundation (Grants tsqn202408113, ZR2023QA075); the Quantum Science and Technology‐National Science and Technology Major Project (Grant 2025ZD0301000); the National Key Research and Development Program of China (Grant 2023YFA1407100); the Guangdong Province Science and Technology Major Project (Future functional materials under extreme conditions—2021B0301030005).

## Supporting information


Supporting Information S1

## Data Availability

The experimental and simulation data that support the findings of this work are available from the corresponding author upon reasonable request.
