# Supplementary material for: Visualization and Control of Quasi‐Monoenergetic Proton Shock Waves in a Single Nanoplasma
Source: Nanophotonics. 2026 Jul 5;15(13):e70198. doi: 10.1002/nap2.70198 (PMC13642470; doi:10.1002/nap2.70198)
Supplement: Supplementary file 1 [file NAP2-15-e70198-s001.docx]

**Title:**

**Visualization and Control of Quasi-monoenergetic Proton Shock Waves in a Single Nanoplasma**

*Zhijie Yang^1,2^, Fenghao Sun^3*^, Guangqi Fan^2^, Jinmei Zheng^4^, M. F. Ciappina^6,7,8^, HuiLi^5^, and Qingcao Liu^2^*

^1^School of Physics, Harbin Institute of Technology, Harbin 150001, China.

^2^College of Science, Harbin Institute of Technology, Weihai 264209, China.

^3^School of Information Science and Engineering, Harbin Institute of Technology, Weihai 264209, China.

^4^School of Marine Science and Technology, Harbin Institute of Technology, Weihai 264209, China.

^5^State Key Laboratory of Precision Spectroscopy, East China Normal University, Shanghai 200241, China.

^6^Department of Physics, Guangdong Technion Israel Institute of Technology, 241 Daxue Road, Shantou, Guangdong 515063, China.

^7^Technion -- Israel Institute of Technology, Haifa, 32000, Israel.

^8^Guangdong Provincial Key Laboratory of Materials and Technologies for Energy.

E-mail: [fhsun@hit.edu.cn](mailto:fhsun@hit.edu.cn); [qingcao.liu@hit.edu.cn](mailto:qingcao.liu@hit.edu.cn)

1. **Experimental data processing**

In a single-shot experiment, each proton momentum distribution (PMD) corresponds to a single nanoparticle excited by an individual laser pulse. Since the focused laser beam exhibits a Gaussian intensity profile and the size of nanoparticles is several orders of magnitude smaller than the laser focus, each nanoparticle will experience different effective intensity depending on its specific position within the laser focus.

In a photoionization experiments, the yields of photoelectrons and photoions approximately linearly increased with laser intensity. Consequently, the acquired spectra effectively map the spatial intensity distribution of the laser focus. In our study, data were sampled exclusively at the center of the focal volume region. To achieve this, we sorted the detected ion yields (excluding background noise) per laser shot under specific conditions (e.g. peak laser intensity 4*I*_0_=4.0 × 10^14^ W/cm^2^ and 100nm gold nanoparticles) and displayed the results in a histogram, as shown in Figure S1. Since the highest-energy photoions originate from ionization events in the central region of the laser focus, a grey region is marked at the end of the histogram.

Single-shot images corresponding to this grey region were selected for subsequent analysis. The single-shot images presented in Figure 2 of the main text were selected from the high-energy photoion data set (see the grey box in Figure S1(a)). Similarly, the peak radial momentum values in Figure 4 were obtained by integrating and averaging the samples within these high-energy photoion sets across different laser intensities, with the calculated standard deviation serving as the error.

Figure S1(b) shows a histogram of ion yields as a function of peak laser intensity, ranging from *I*_0_=1.0 × 10^14^ W/cm^2^ to 4*I*_0_=4.0 × 10^14^ W/cm^2^. The histograms differ across the three effective laser intensities. Both the photoion yield and the cutoff energy increase monotonically with laser intensity. Additionally, as the intensity grows, the focal volume region where ionization occurs also expands.

**Figure S1.** Histograms of ion yields. (a) The counts of laser shot events with ion yields detected for 100 nm Au nanoparticles at 4*I*_0_=4.0 × 10^14^ W/cm^2^. The inset shows a cross-sectional schematic of a Gaussian focus, illustrating the focal volume region with varying intensities. (b) A similar histogram for the same particle size but for three different laser intensities (*I*_0_=1.0 × 10^14^ W/cm^2^).
